# Versatility of the Mec1^ATM/ATR^ signaling network in mediating resistance to replication, genotoxic, and proteotoxic stresses

**DOI:** 10.1007/s00294-018-0920-y

**Published:** 2019-01-05

**Authors:** Isaac Corcoles-Saez, Kangzhen Dong, Rita S. Cha

**Affiliations:** School of Medical Sciences and North West Cancer Research Institute, Bangor University, Bangor, LL57 2UW UK

**Keywords:** ATM/ATR, Mec1, Rad53, Dun1, Sml1, Proteotoxic stress, Proteostasis, Heavy metals, Amino acid analogues, Huntingtin, Checkpoint kinases

## Abstract

The ataxia-telangiectasia mutated/ATM and Rad3-related (ATM/ATR) family proteins are evolutionarily conserved serine/threonine kinases best known for their roles in mediating the DNA damage response. Upon activation, ATM/ATR phosphorylate numerous targets to stabilize stalled replication forks, repair damaged DNA, and inhibit cell cycle progression to ensure survival of the cell and safeguard integrity of the genome. Intriguingly, separation of function alleles of the human *ATM* and *MEC1*, the budding yeast *ATM*/*ATR*, were shown to confer widespread protein aggregation and acute sensitivity to different types of proteotoxic agents including heavy metal, amino acid analogue, and an aggregation-prone peptide derived from the Huntington’s disease protein. Further analyses unveiled that ATM and Mec1 promote resistance to perturbation in protein homeostasis via a mechanism distinct from the DNA damage response. In this minireview, we summarize the key findings and discuss ATM/ATR as a multifaceted signalling protein capable of mediating cellular response to both DNA and protein damage.

## Introduction

The maintenance of protein homeostasis or proteostasis is crucial for cellular function and survival. Key processes that impact proteostasis include protein translation, modification, trafficking, and degradation. In addition, the protein quality control (PQC), an ancient cytoprotective mechanism for minimizing misfolded, damaged and aggregated proteins play a crucial role in safeguarding integrity of the cellular proteome (Díaz-Villanueva et al. 2015; Hill et al. 2017). In humans, deficits in proteostasis are linked to a range of diseases including neurodegeneration and cancer (Kurtishi et al. 2018; Van Drie 2011). Currently, relatively little is known about the ways in which perturbation in proteostasis is sensed and signaled. Here, we discuss recent findings implicating the ATM/ATR DNA damage response (DDR) network in mediating cellular response to proteotoxic stress.

## Emergence of DDR-independent functions of ATM/ATR kinases

ATM (ataxia-telangiectasia mutated) and ATR (ATM and Rad3-related) are serine/threonine kinases belonging to the phosphatidylinositol 3-kinase-related kinases (PIKKs) superfamily (Lovejoy and Cortez 2009). ATM/ATR proteins are found in all eukaryotes examined to date, including *Saccharomyces cerevisiae* (Tel1/Mec1), *S. pombe* (Tel1/Rad3), *D. melanogaster* (Tel1/Mei41) and *A. thaliana* (ATM/ATR) (Hari et al. 1995; Greenwell et al. 1995; Bentley et al. 1996; Elledge 1996; Garcia et al. 2000). These proteins orchestrate the DNA damage response (DDR) in the respective organism, a highly complex and interconnected set of processes that safeguard integrity of the genome and promote survival of the cell in response to DNA damage or perturbation in genome duplication (Harper and Elledge 2007; Moriel-Carretero et al. 2018).

At the cellular level, loss of ATM/ATR function results in genome instability and acute sensitivity to genotoxic agents. In humans, inactivation of ATM or ATR leads to ataxia-telangiectasia (A-T) or Seckel syndrome, respectively, a rare autosomal recessive disease characterized by a constellation of symptoms including cerebellum ataxia, cancer, diabetes, growth retardation and/or microcephaly (O’Driscoll et al. 2003; Llorens-Agost et al. 2018). While deficits in the DDR underpin some of these conditions, they do not adequately account for all clinical manifestations of A-T and Seckel syndrome. Therefore, it was not surprising when evidence for DDR-independent functions of ATM/ATR proteins (e.g., glucose metabolism and neuronal vesicle trafficking) began to emerge (Dahl and Aird 2017; Cheng et al. 2018; Botchkarev and Haber 2018; Harari and Kupiec 2018).

## Essential function(s) of Mec1 in proteostasis

**Fig. 1:**
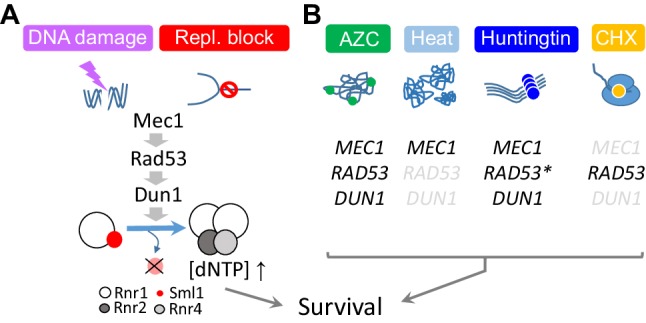
Essential roles of the Mec1 signaling network in mediating resistance to replication, genotoxic, and proteotoxic stresses. **a** The canonical Mec1-dependent DDR. DNA damage and replication stress, exemplified by a DNA double strand break (DSB) and replication block, respectively, activate the Mec1–Rad53–Dun1 signaling cascade. Sml1 is an allosteric inhibitor of Rnr1, the major catalytic subunit of budding yeast RNR, which comprise a Rnr1 homodimer, Rnr2, and Rnr4. The Mec1–Rad53–Dun1-dependent destruction of Sml1 promotes de novo dNTP synthesis necessary for genome duplication and DNA damage repair. **b** Differential requirement of *MEC1, RAD53*, and *DUN1* in mediating resistance to AZC, heat, Htt103Q, and CHX (Corcoles-Saez et al. 2018). Genes shown in black are required for survival. Genes shown in light grey are dispensable. *Not tested

Structurally, budding yeast Mec1 and Tel1 resemble the mammalian ATR and ATM, respectively. However, Mec1 performs most functions of both ATR and ATM, while deletion of *TEL1* does not confer an obvious phenotype (Weinert et al. 1994; Mallory and Petes 2000). In response to DNA damage or replication stress, Mec1 phosphorylates Rad53, an essential effector kinase and an ortholog of the mammalian CHK1. Activated Rad53, in turn, phosphorylates Dun1 (Chen et al. 2007). The Mec1–Rad53–Dun1 signaling cascade increases dNTP production via Dun1-phosphorylation-dependent destruction of Sml1, an allosteric inhibitor of Rnr1, the major catalytic subunit of the budding yeast ribonucleotide reductase (RNR) (Zhao et al. 1998, 2001; Chabes et al. 1999) (Fig. 1a). The Mec1, Rad53, and Dun1-dependent removal of Sml1 and the ensuing increase in dNTP abundance is crucial for accurate repair of damaged DNA and survival of the cell (Zhao et al. 2001).

Mec1 is required for viability. The fact that Sml1 inactivation bypasses this requirement and that Mec1 promotes Sml1 degradation at the onset of S phase (Zhao and Rothstein 2002; Earp et al. 2015) suggests that an essential Mec1 function is to activate dNTP production necessary for genome duplication (Zhao et al. 1998, 2001; Zhao and Rothstein 2002). To confirm the hypothesis, we directly assessed the impact of Mec1-inactivation on dNTP abundance utilizing *mec1-4*, a conditional lethal allele (Cha and Kleckner 2002; Earp et al. 2015). Surprisingly, the extent of reduction in dNTP pool was insufficient to account for *mec1* lethality, implying that the cell death was attributable to a different defect(s) (Earp et al. 2015).

To gain a fuller understanding of Mec1’s functional repertoire, we performed synthetic genetic array (SGA) analysis of *mec1-4*, the above-mentioned conditional lethal allele (Corcoles-Saez et al. 2018). SGA is a high throughput technique for identifying genetic interactors of a gene of interest (Baryshnikova et al. 2010). As expected, the screen identified numerous genes involved in well-established function of Mec1, for example, DNA damage checkpoint response, genome duplication, and DNA recombination. In addition, the screen identified a number of novel *mec1* interactors, including those involved in proteostasis, such as *JJJ3*, encoding for a member of the Hsp40/DnaJ family of molecular chaperones (Walsh et al. 2004), *TIM18* involved in mitochondrial protein homeostasis (Kerscher et al. 2000), and *KTI12* required for tRNA modification and protein translation (Fichtner et al. 2002). Furthermore, the *mec1-4* mutation conferred acute sensitivity to different types of proteotoxic stresses including; (1) azetidine-2-carboxylic acid (AZC), a proline analogue, which induces protein misfolding upon incorporation into nascent polypeptides (Weids and Grant 2014). (2) Htt103Q, an aggregation-prone poly glutamate (polyQ) model peptide derived from the Huntingtin’s disease protein (Meriin et al. 2002). And (3) heat, which induces widespread protein denaturation and misfolding. The *mec1-4* lethality caused by AZC, Htt103Q or heat was accompanied by widespread protein aggregation, and autophagy activation rescued the lethality by facilitating aggregate resolution (Corcoles-Saez et al. 2018). Remarkably, *sml1Δ* also rescued the temperature- and AZC-sensitivity of *mec1-4* cells by minimizing the steady-state aggregate level, implicating a role of Sml1 in proteostasis. In further support, we found that genetic interactors of *SML1* identified by SGA analysis were enriched for genes involved in protein translation (Costanzo et al. 2010; Corcoles-Saez et al. 2018).

Intriguingly, the *mec1-4* mutation confers robust resistance to cycloheximide (CHX), a potent inhibitor of protein synthesis (Corcoles-Saez et al. 2018). The *mec1-4* phenotype (i.e., resistant to CHX and sensitive to heat) is reminiscent of a group of mutants collectively referred to as cycloheximide-resistant, temperature sensitive lethal (*crl*) mutants (McCusker and Haber 1988a, b). A subsequent study found that majority of the *crl* mutations reside in the genes encoding for a component of the proteasome (Gerlinger et al. 1997). The phenotypic similarities between the *crl* and *mec1-4* mutants suggest that the *mec1-4* mutation might also impact proteosome function.

## Genotoxic- versus proteotoxic-stress response pathways

During the DNA damage- or replication stress-checkpoint response, Mec1, Rad53, and Dun1 work together as a functional unit. As such, inactivation of each impairs Sml1 removal and confers sensitivity to both MMS and HU (Zhao et al. 2001). In contrast, we find that *mec1, rad53*, and *dun1* mutants exhibit a unique sensitivity profile against different types of proteotoxic stresses (Corcoles-Saez et al. 2018) (Fig. 1b). *mec1-4* cells are sensitive to heat, AZC, and Htt103Q but resistant to CHX. Similarly, *dun1*Δ cells exhibit sensitivity to AZC and Htt103Q and resistance to CHX; however, unlike *mec1-4, dun1*Δ cells are resistant to heat. *rad53-K277A*, a kinase dead allele impaired in the DDR, differs from both *mec1-4* and *dun1*Δ in that it confers acute sensitivity to CHX (Fig. 1b). These results indicate that the Mec1-dependent survival under each condition is mediated via a distinct pathway. The human ATM is also shown to promote resistance to proteotoxic stress by a DDR-independent mechanism (Lee et al. 2018).

## Limited “target specificity” of replication, genotoxic and proteotoxic stresses

Several studies have shown or suggested a crosstalk between the DDR and proteostasis. For example, the Mec1–Rad53–Dun1 signaling cascade promotes survival in response to cadmium, a proteotoxic metal, and perturbation in the copper or iron homeostasis (Dong et al. 2013; Baek et al. 2012; Sanvisens et al. 2016). In yeast, HU or MMS exposure leads to changes in the location and/or abundance of proteins involved various aspects of proteostasis, including protein translation, folding and degradation (Tkach et al. 2012). Mec1, Rad53, and the human ATM/ATR phosphorylate proteins involved in protein homeostasis in response to genotoxic stress (Matsuoka et al. 2007; Zhou et al. 2016). It was also shown that pre-mRNA splicing factors play a role in detecting, signaling, and repairing damaged DNA (Mikolaskova et al. 2018).

The crosstalk between the DDR and proteostasis is not unexpected given that most genotoxic and replication stress-inducing agents exert profound impact on proteostasis. For example, HU, a widely utilized replication stress-inducing agent, is a radical scavenger that inhibits dNTP production by extracting a critical iron from the active site (Nyholm et al. 1993). Evidence indicates that HU cytotoxicity is linked to perturbation in proteostasis and remarkably, can be uncoupled from its impact on RNR (Davies et al. 2009; Liew et al. 2016). Furthermore, molecular chaperons Hsp70 and Hsp90, key components of the PQC, impact RNR function (Knighton et al. 2018). MMS, a popular genotoxic agent, covalently modifies amino acids and perturbs cellular redox homeostasis, which in turn impacts protein folding and function (Gasch et al. 2001; Jiang et al. 2012). Ionizing radiation, another widely utilized genotoxic agent, is a potent proteotoxic agent (Radman 2016). Proteotoxic agents (e.g., heavy metals) may also exert a profound impact on genome stability by perturbing the proteostasis of proteins involved in genome maintenance, including the RNR and DNA polymerases α, δ, and ε, whose function depends on iron homeostasis (Nyholm et al. 1993; Stehling et al. 2012).

## Conclusion and perspectives

Given the apparent lack of target specificity among genotoxic and protoetoxic agents, it would make an economical sense for a cell to utilize the same signaling network to respond to both DNA and protein damage. Evidence suggests that the ATM/ATR signaling network fulfills this function. Several outstanding questions remain, including the molecular mechanism(s) by which ATM/ATR proteins sense and signal proteotoxic stress and restore proteostasis. Importantly, the multifaceted nature of the ATM/ATR signaling provides a new conceptual framework in understanding the complex cellular phenotypes and clinical manifestations associated with ATM/ATR inactivation.
